# *In vivo* evolution of tumour cells after the generation of double-strand DNA breaks

**DOI:** 10.1038/sj.bjc.6600959

**Published:** 2003-05-27

**Authors:** H Mekid, O Tounekti, A Spatz, M Cemazar, F Z El Kebir, L M Mir

**Affiliations:** 1 Vectorologie et transfert de gènes, UMR 8121 CNRS, Institut Gustave-Roussy, 39 rue C. Desmoulins, F-94805 Villejuif Cédex, France; 2 Laboratoire de Biochimie et Technobiologie, Faculté des Sciences de Tunis, Campus Universitaire, 1060 Tunis, Tunisia; 3 Département d'Anatomie Pathologique, Institut Gustave-Roussy, Villejuif 94805, France; 4 Department of Tumor Biology, Institute of Oncology, Ljubljana, Slovenia; 5 Laboratoire de Biologie du Développement, Université d'Oran Es Sénia, 31000 Oran, Algeria

**Keywords:** apoptosis, mitotic cell death, bleomycin, electrochemotherapy, electroporation, *in vivo* electropermeabilization

## Abstract

*In vitro*, the ratio of single- to double-strand DNA breaks (DSB) and their absolute values determine the cell death pathway. The consequences of the generation of various numbers of DSB generated *in vivo* in tumour cells have been analysed in two different experimental tumour models. Synchronisation of DSB generation and control of their number have been achieved using different doses of bleomycin (BLM) and tumour cell permeabilisation by means of locally delivered electric pulses. According to BLM dose, different cell death pathways are observed. At a low therapeutic dose, a mitotic cell death pathway is detected. It is characterised by the appearance of ‘atypical mitosis’, TUNEL and caspase-3 positive, 24 h after the treatment, and later by the presence of typical apoptotic figures, mainly TUNEL positive but caspase-3 negative. Caspase-3 is thus an early marker of apoptosis. Mitotic cell death is also followed by lymphocytic infiltration reaction. At high doses of BLM, pseudoapoptosis is detected within a few minutes after the treatment. These cell death pathways are discussed as a function of the number of DSB generated, by comparison with previous results obtained *in vitro* using BLM or ionising radiation.

Cell death following exposure to toxic agents can occur through several routes, including apoptosis, extended or permanent cell cycle arrest, necrosis and ‘mitotic catastrophe’. These various types of cell death display different morphological changes and biochemical characteristics. Such variations can result from differences between cell types and the mechanisms by which cell death is triggered.

At the cellular level, necrosis is characterised by an increased plasma membrane permeability, cell swelling, a decline in protein synthesis and autolysis (Wyllie *et al*, 1980; Schwartz and Osborne, 1993). It can be observed in solid tumours of mice treated with arsenic trioxide (Lew *et al*, 1999).

Apoptotic cell death plays a major role in the regulation of cell growth in multicellular organisms. It constitutes a systematic means of cell suicide during normal embryonic development and morphogenesis (von Wangenheim and Peterson, 1998), aging (Monti *et al*, 1992) or in response to pathogenic infections and other irreparable cell damage. Induction of apoptosis by anticancer drugs has been demonstrated using, for example, paclitaxel (Milross *et al*, 1996) or cisplatin (Trimmer and Essigmann, 1999). Recently, after cell treatment with ionising radiation, a ‘premitotic’ and a ‘postmitotic’ apoptosis could be distinguished as a function of the morphological features of the treated cells (Shinomiya *et al*, 2000). Apoptosis requires the activation of proteases and endonucleases (Wyllie *et al*, 1984; Anderson *et al*, 1997). However, Tounekti *et al* (1993) showed that, *in vitro*, cell uptake of large quantities of BLM, a nonpermeant drug that generates double-strand DNA breaks (DSB), results in the very rapid generation of the apoptotic morphological changes, as well as in a DNA degradation similar to that observed in usual apoptosis. This evolution was termed pseudoapoptosis (Tounekti *et al*, 1993) because it is caused by the DSB generated by the bleomycin (BLM) and does not require induction of proteases nor endonucleases involved in typical apoptosis: BLM itself acts as a microendonuclease (Tounekti *et al*, 1995).

*In vitro*, mitotic cell death is a slow process in which cell metabolism remains functional for a period equivalent to more than two cell cycles, the cell death being noticeable only after the passage of the cell through one or more consecutive mitoses (Chang and Little, 1991; Radford, 1991). These mitoses are, nevertheless, aberrant and do not result in the formation of two daughter cells (Dewey *et al*, 1970; Joshi *et al*, 1982). This results in the generation of small abortive colonies by otherwise clonogenic cells. Mitotic cell death can be initiated by the generation of a few unrepaired DSB like those generated by small doses of ionising radiation or the internalisation of low amounts of radiomimetic drugs like BLM (Tounekti *et al*, 1993, 2001; Yanagihara *et al*, 1995).

Thus, *in vitro*, the number of DSB seems to determine the cell death pathway. Single-strand DNA breaks (SSB) in very large numbers also result in cell death (Yoshida *et al*, 1993; Cohen-Jonathan *et al*, 1999). We also reported that not only the absolute numbers of DSB or SSB but also their ratio determine cell death pathway: true apoptosis, pseudoapoptosis or mitotic cell death (Tounekti *et al*, 2001).

On these basis, we decided to perform a systematic analysis of the consequences of the generation of different numbers of DSB in tumour cells *in vivo*. Control of DSB number, as well as synchronisation of the DSB generation was achieved using different doses of BLM and cell permeabilisation by means of locally delivered electric pulses (EPs). Indeed, (a) external electric fields generate changes in the transmembrane voltage and, under appropriate conditions, provoke the transient and reversible permeabilisation of the cells, *in vitro* as well as *in vivo* and (b) BLM molecules do not diffuse through the plasma membrane and they enter only at the time of the cell electropermeabilisation, in amounts proportional to the external concentration at the moment of EP. Moreover, permeabilising EP can be applied locally *in vivo* on tumour nodules: the huge increase in the antitumour effectiveness of BLM achieved by the increase in BLM uptake is the basis of electrochemotherapy (ECT), a new way to treat solid tumours that is already under clinical evaluation (Domenge *et al*, 1996; Mir *et al*, 1998; Gehl and Geertsen, 2000; Rodriguez-Cuevas *et al*, 2001; Rols *et al*, 2002). Therefore, it seemed possible to study *in vivo* the cell death pathway caused by different amounts of DSB using these experimental conditions. Indeed, in ECT, as performed in our preclinical and clinical studies, EPs alone are not toxic and the toxicity observed is actually the toxicity caused by the facilitated uptake of BLM.

## MATERIALS AND METHODS

### Tumour cells culture and tumour production

The LPB cell line, a methylcholanthrene-induced C57Bl/6 mouse sarcoma cell line (Belehradek *et al*, 1972), and the B16 F0 melanoma cells (ATCC CRL 6322) were cultured using classical procedures and minimum essential medium culture medium (Gibco BRL, Cergy-Pontoise, France) supplemented with 100 U ml^−1^ penicillin, 100 mg ml^−1^ streptomycin (Sarbach, France) and 8% fetal calf serum (Gibco). These two cell lines had been previously used in the laboratory in preclinical trials of the ECT (Mir *et al*, 1991, 1992; Sersa *et al*, 1994) as well as for analysing *in vivo* cell electrofusion (Mekid and Mir, 2000). C57Bl/6 female mice, 6–8 weeks old, were inoculated subcutaneously in the left flank with 1 × 10^6^ to 1.2 × 10^6^ syngeneic LPB cells or B16 cells, producing tumours 6–7 mm of diameter 9 days later. Mice were anaesthetised using a mixture of xylazine 12.5 mg kg^−1^ (Bayer Pharma, Puteaux, France) and ketamine 125 mg kg^−1^ (Parke Davis, Courbevoie, France). Animals were housed and handled according to the recommended guidelines (Workman *et al*, 1998). They were humanely killed by CO_2_ inhalation.

### Tumour treatment

Bleomycin (Roger Bellon, Neuilly, France) was dissolved in sterile 0.9%. NaCl, aliquoted and stored at −20°C. At 4 min before EP delivery, BLM was injected in the retro-orbitary sinus at a dose of either 10 *μ*g, 100 *μ*g or 1 mg in 100 *μ*l NaCl, per mouse, (approximately 0.5, 5 or 50 mg kg^−1^).

Tumour electropermeabilisation was performed as previously described (Mir *et al*, 1991). Stainless-steel external plate electrodes were placed on both sides of the protruding tumour, contact with skin was ensured by electrocardiography paste. Square-wave EPs (eight pulses of 100 *μ*s delivered at a frequency of 1 Hz) were generated by a PS15 electropulsator (Jouan, St Herblain, France) and controlled through a VC-6025 oscilloscope (Hitachi, Japan). For 1350 V cm^−1^ pulses, 800 V was applied between two parallel electrodes 10 mm large and 6 mm apart. It was already known that these electrical parameters allow for the *in vivo* reversible electropermeabilisation of almost of cells in the LPB and B16F0 tumours since the administration of these EPs alone, without BLM, (like the administration of the BLM alone, with no EP), caused no statistically significant tumour growth delay (with respect to the control untreated tumours), while the concomitant administration of these EPs with BLM resulted in the achievement of complete regressions and even of cures (Mir *et al*, 1991, 1992, 1996; Sersa *et al*, 1994; Mekid and Mir, 2000). Then, mice were returned to their cages for different periods (between 1 and 100 h) and killed to remove the tumours for histological processing. Three mice (i.e. three independent tumours) were used for each experimental condition.

### Histological procedures

Tumours were fixed in AFA (75% ethanol, 5% acetic acid and 2% of formaldehyde 40%) for 24 h, dehydrated and included in paraffin. Slices of 5 *μ*m were prepared using a Reichert-Jung 2030 microtome (Microm-Zeiss, Jena, Germany). Then slides were rehydrated and stained with HES (hemalun, 0.2%; eosin, 0.3%, saffron, 5%). Alternatively, rehydrated slides were washed in phosphate-buffered saline (PBS; 50 mM Na_2_HPO_4_, 50 mM NaH_2_PO_4_ and 200 mM NaCl, pH 7.4) and stained with 3 *μ*M propidium iodide (PI) (Sigma, La Verpillière, France).

### Immunohistochemical cell death determinations

Apoptosis-specific staining (based on free DSB extremities labelling) was performed using the In-situ-Cell-Detection Kit, either alkaline phosphatase (AP) or fluorescein (Roche Molecular Biochemical, France). Deparaffinated slides were washed in PBS, digested with 20 *μ*g ml^−1^ Proteinase K (Sigma) and processed according to the manufacturer's instructions. Briefly, free 3′-OH termini were labelled with fluorescein isothiocyanate (FITC)-labelled deoxyuridine using the terminal transferase enzyme and detected either by fluorescence microscopy or with an AP-coupled anti-fluorescein antibody, and the subsequent incubation with fast red substrate.

The implication of a caspase-dependent cell death pathway was studied using an anti-activated caspase-3 antibody (Pharmingen, San Diego, USA) (Hadjiloucas *et al*, 2001). Slices were deparaffinated, treated in a microwave oven and incubated overnight at 4°C in the presence of the 1 : 500 diluted antibody. The positive cells were revealed using an appropriate Power Vision Detection AP System (ImmunoVision Technologies, Dalycity, CA, USA) and analysed using a Leica DMRB light microscope (Leitz, Wetzlar, Germany).

### Electron microscopy

The treated tumours were fixed with phosphate-buffered glutaraldehyde (Ladd Research Industries, England), postfixed with osmium tetroxide (Prolabo, France) and then dehydrated, processed, sliced and observed under a Zeiss 902 electron microscope.

### Microscopic slides examination

Slides were examined under a Leica DMRB microscope equipped with an automatic photographic device. For fluorescence microscopy, the filter set for rhodamine detection was used to observe the PI-stained slices and the filter set for FITC detection to observe the apoptotic cells stained with the In-situ-Cell-Detection Fluorescein Kit.

For each experimental condition, three mice were treated, and from each mouse, three HES-stained tumour slides were prepared. In all the cases, the entire slide was examined to detect all the types of cell patterns generated under each experimental condition. To determine the percentages of each cell pattern, all the cells from four randomly chosen fields of non-necrotic areas of each of the three slices prepared from each tumour were counted. The percentages of each cell pattern were calculated with respect to the total number of cells counted. On the average, 1500 cells were counted for each experimental condition.

### Statistical analysis

All data are presented as means and standard deviations. Statistical analysis of the significance of the difference between the controls and the groups treated under the various experimental conditions was performed using Student's *t*-test.

## RESULTS

Based on their morphology, tumour cells were classified into four categories: (a) cells with normal morphology and size, (b) atypical cells (see below), (c) apoptotic cells showing classical DNA condensation and (d) typical mitotic cells. The percentages of cells in each category were determined at various times after tumour exposure to EP in BLM-injected mice (i.e. after ECT). Morphological estimation of apoptosis was based on cell characteristics, such as marked condensation and marginalisation of the chromatin or formation of apoptotic bodies. Atypical cells were the ones showing extended and intense pycnosis, where it was impossible to distinguish between the nucleus and cytoplasm. Under some conditions, further described, cells with normal morphology but larger than those found in untreated tumours were also observed. Even though their apparent diameter could reach twice the apparent diameter of the normal cells, they were still counted as cells with normal morphology.

### Histological characteristics of the untreated tumours

**Figure 1:**
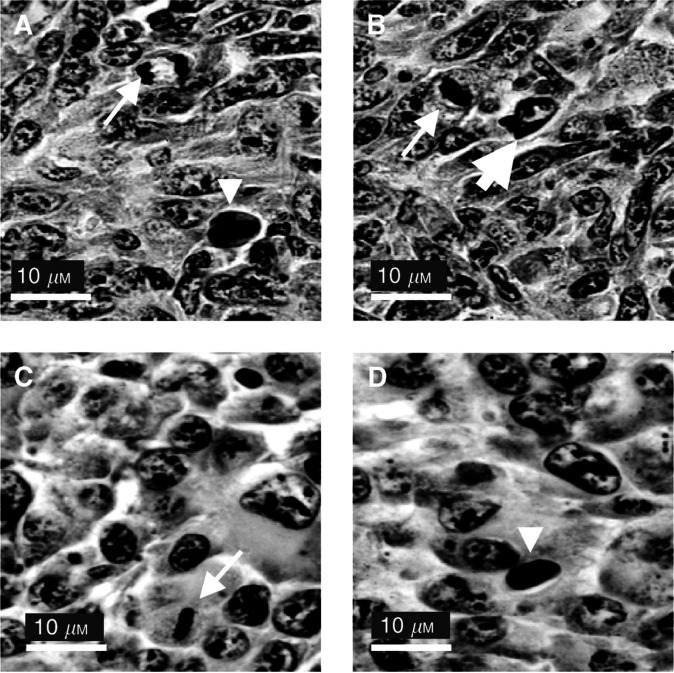
Histological characteristics of the untreated murine fibrosarcoma tumour LPB (**A** and **B**) and murine melanoma tumour B16F0 (**C** and **D**). Small arrows: mitotic cells; large arrows: apoptotic cells; arrowheads: atypical cells.

**Figure 2:**
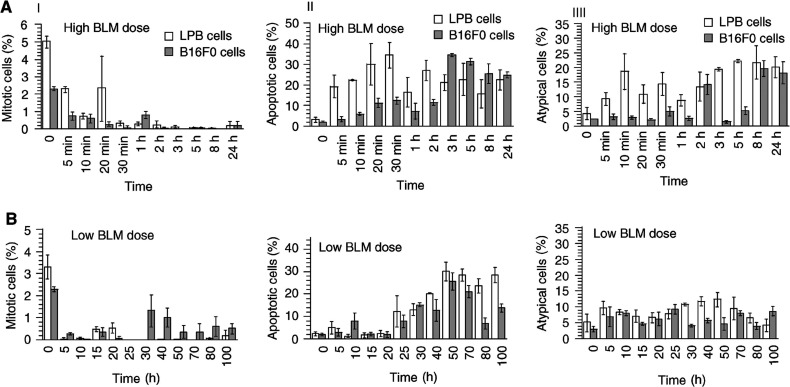
Kinetics of the fraction of (**I**) mitotic cells, (**II**) apoptotic cells and (**III**) atypical cells in LPB and B16F0 tumours after ECT using 1 mg BLM (panels A) or 10 *μ*g BLM (panels B). Statistical analysis: all groups were compared to the respective controls by means of Student's *t*-test. All groups were significantly different from the controls (at least *P*<0.05) except in panels **IIA** LPB (at 1 and 8 h), **IIA** B16F0 (at 5 min and 1 h), **IIB** LPB (before 20 h), **IIB** B16F0 before 25 h, **IIIA** B16F0 (at 5 min and 1 h), **IIIA** LPB (20 min, 1 h and 2 h), **IIIB** B16F0 at 50 h and **IIIB** LPB at 15, 20, 25 and after 70 h.

As revealed by HES staining, LPB fibrosarcoma was a tissue in which many mitosis were detectable (Figure 1A, B

). Cells were densely packed and the membrane separating each individual cell was not always distinguishable. In the absolute control 5±0.3% of cells were in mitosis (Figure 2I

), 2.7±0.1% in the EP alone control and 3±0.2% in the BLM alone control (the data concerning the EP alone control and the BLM alone control are not shown in the figures; they were obtained on tumours removed and fixed 5 h after the respective treatment). The percentages of typical apoptotic figures (Figure 1B) were 3.1±0.9% in the absolute control (Figure 2II), 2.7±0.7% in the EP and 2.5±0.5% in the BLM controls. Atypical figures (Figure 1A) were regularly found in all LPB control tumours: 4.3±1.8% in the absolute control (Figure 2III), 5.5±0.7% in the EP and 5±0.7% in the BLM controls.

In the B16 tumours, the membrane limiting each individual cell was clearly visible (Figure 1C, D). The nuclei in the untreated control tumours displayed a relatively regular size and were usually located in the centre of the tumour cells. In absolute control, 2.3±0.1% of the cells were in mitosis (Figures 1C and 2I), 4.0±0.1% in the EP alone and 1.3±0.1% in the BLM alone controls. The percentages of typical apoptotic figures were 2.0±0.2% in the absolute control (Figure 2II), 3.5±0.3% in the EP and 2.6±0.5% in the BLM controls. Atypical figures (Figure 1D) were regularly found also in all the B16F0 control tumours (2.4±0.1% in the absolute control (Figure 2III), 4.0±0.6% in the EP and 7.0±0.7% in the BLM controls).

No immune cell infiltrated the LPB tumour tissue in the absence of treatment. Some lymphocytes were detected at the periphery of the tumour only. In the B16 melanoma, spontaneous necrosis areas were found even in small-untreated tumours. In these areas, immune cells could be easily observed (data not shown).

### Changes in the fraction of cells in mitosis after *in vivo* exposure to BLM and EP

#### Using a high dose of BLM (1 mg per mouse)

After the treatment, a rapid decrease in the fraction of cells in mitosis was observed in both tumours, and after 3 h mitoses became almost undetectable (Figure 2IA). At 5 min after the treatment of LPB or B16F0, the percentage of mitosis decreased to approximately 40–45% of controls. These percentages continued to decline significantly in the tumours at 1 h, and later, after the ECT (Figure 2IA). Interestingly, the existence of abnormal mitosis was detected in HES-stained slices 10–20 min after the treatment. At 30 min, all the mitoses still visible displayed aberrant figures. No mitosis was observed thereafter.

#### Using a low dose of BLM (10 μg per mouse)

A similar drastic reduction of the fraction of cells in mitosis was observed in both the tumours (Figure 2IB). The percentage of mitosis in LPB and B16F0 was found to be reduced at 5 h after the treatment and remained at this low value until 100 h in the case of the LPB tumour. In the case of B16F0, the decrease was smaller at 5 h than the decrease observed in LPB, and some cells in mitosis were still detected at 30 h. However, these percentages were lower than in untreated controls (Figure 2IB).

### Changes in the fraction of apoptotic cells

#### Using 1 mg BLM per mouse

In the LPB tumours, cell morphology started to change in certain areas of the tumours very shortly after the EP delivery. As revealed by the HES staining, as soon as 5 min after the treatment, 20% of the observed nuclei displayed condensation of chromatin that evoked apoptosis. At 10 min, the percentage was 10-fold larger (22.4±0.3%) than that observed in control tumours. A maximum of 34.5±6.3% of nuclei with condensed and margined chromatin was reached at 30 min (Figure 2IIA).

**Figure 3:**
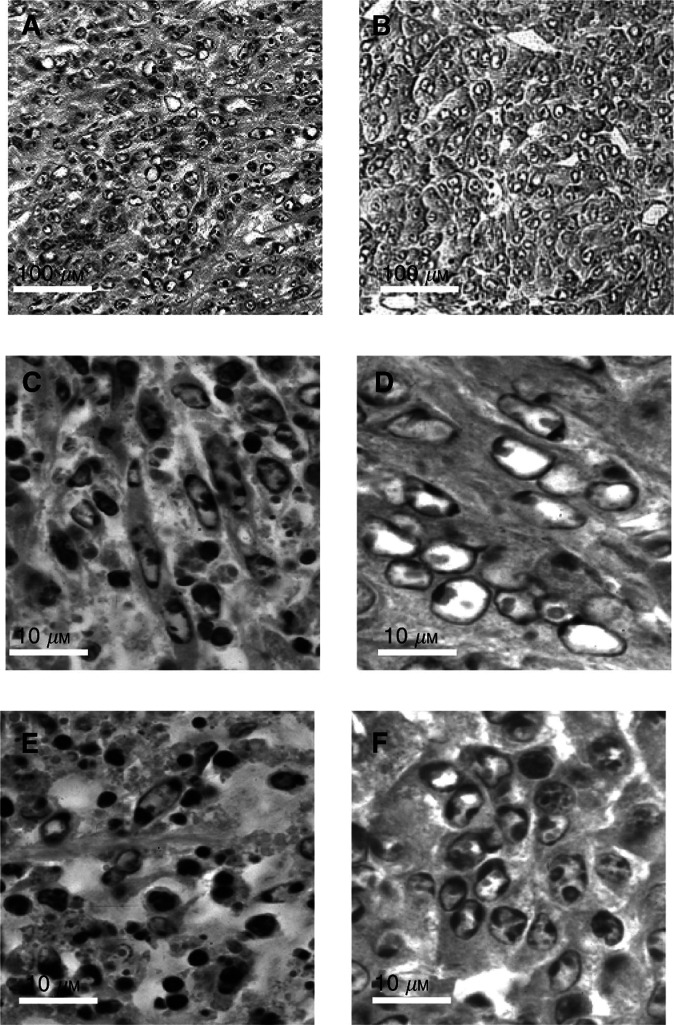
Histological changes observed in tumours removed 30 min after ECT using 1 mg BLM (**A**–**D**) and in tumours removed 50 h after ECT using 10 *μ*g BLM (**E** and **F**); (**A**, **C** and **E**): LPB tumours; (**B**, **D** and **F**): B16F0 tumours.

Changes were more pronounced in certain areas of the tumours slices where all the cells displayed marked condensation of the chromatin, and sometimes already the appearance of apoptotic bodies (Figure 3A, C

). In order to clearly visualise the condensation and marginalisation of chromatin in nuclei and to confirm these data, we also stained some slices with PI (data not shown).

In the B16F0 tumours treated under the same conditions, a massive percentage of apoptotic cells were also observed in some areas of the tumour (Figure 3B, D). However, the increase in apoptotic cells was more progressive (Figure 2IIA) since a highly significant five-fold increase was observed only at 20 min (11.3±2.3%). For both LPB and B16F0 tumours, a decrease in the fraction of the apoptotic cells was observed at 1 h after the treatment, followed, respectively at 2 and at 3 h, by an ulterior increase (Figure 2IIA).

#### Using 10 μg BLM per mouse

In the presence of a low amount of BLM, it was possible to subdivide the evolution of the fraction of apoptotic cells into two intervals (Figure 2IIB), before and after 25 h after the ECT. In the interval of 0–25 h, the percentage of apoptotic cells was similar to that of the control tumours (around 2%), although a significant four-fold increase in apoptotic cells was observed in the B16F0 population at 10 h. In both tumours from 25 h until 100 h, the percentage of apoptotic cells progressively increased up to a maximum observed at 50 h (respectively 30.1% for the LPB and 25.6% for the B16F0 tumours) (Figure 2IIB and 3E, F).

In the B16F0 melanoma tissue, it was necessary to distinguish between the areas with individual cells and those with large polykaryotic cells resulting from the *in vivo* electrofusion of B16F0 cells (Mekid and Mir, 2000). In the areas containing individual unfused cells, no change in the percentage of apoptotic cells was found at any time after the treatment between the treated and controls. On the contrary, in the areas displaying fused cells, the percentage of apoptotic cells increased significantly from 25 h after the treatment until 100 h. At 50 h, 92.8% of the whole of the apoptotic nuclei were found in the fused cells, and 96.4% of the nuclei in the fused cells were apoptotic. (data not shown in the figure).

Thus, our results show that apoptotic cells can be found either very rapidly after the treatment in the presence of a high amount of BLM or after more than 25 h in the presence of a low amount of BLM.

### Changes in the fraction of atypical cells

#### Using 1 mg BLM per mouse

A net increase in the fraction of atypical cells (two-fold with respect to the untreated tumours) was found in LPB fibrosarcoma 5 min after the treatment. At 10 min, the increase was approximately four times. This increase persisted at 3 h and later on (at 5, 8 and 24 h) (Figure 2IIIA). Contrary to LPB fibrosarcoma, the percentage of atypical cells became larger (seven to nine times higher) than that observed in untreated control tumours only at 2 h and then at 8 and 24 h, while in the interval between 0 and 1 h, values were close to that of control B16F0 tumours (Figure 2IIIA).

#### Using 10 μg BLM per mouse

The rate of atypical cells in LPB tumours was slightly increased at all times except at 100 h. A significant increase was found at early times (up to 5 h) as well as at later times (30–50 h). This evolution was very different compared to the changes in apoptotic cells in the same fibrosarcoma (Figure 2IIB, IIIB). Contrary to LPB fibrosarcoma, the trend of the changes in the percentage of atypical cells in B16F0 tumours was not clear. Variations were observed from each time point to the next one. Variations with respect to control values never exceeded three times (Figure 2IIIB).

### Other histological changes observed

#### Changes in tumour cell size

At the high dose, no change in cell size was observed: both the atypical and the apoptotic nuclear changes were detected in cells displaying the same apparent diameter than in the untreated tumours. At the low dose, for the first 25 h following the EP, the cells showing a normal nucleus became progressively larger than in untreated tumours. At the end of this period, the apparent diameter of the nuclei could be twice as large as that of the normal cells. This evolution was observed in both LPB and B16F0 tumours treated using 10 *μ*g BLM (data not shown). As a consequence, atypical cells displayed an apparent diameter larger than that of the control tumour cells, while apoptotic cells displayed a diameter lower than that of the atypical cells but still similar to that of the cells in control tumours.

#### Changes in immune cell distribution

After ECT using 1 mg BLM, no change in the distribution of the immune cells infiltrate was observed. Using 10 *μ*g BLM, it was possible to detect a large infiltration of the LPB tumours by lymphocytes from 25 h after the EP delivery. At longer times, such as 50 or 70 h, a large number of infiltrating lymphocytes were present in all the parts of the LPB tumours, but with a higher density where a large fraction of apoptotic cells was also present. In the case of the B16F0 tumours, in which lymphocytes were already detectable in the spontaneous necrosis area controls, no major change could be noticed at any time.

### Electron microscopy

**Figure 4:**
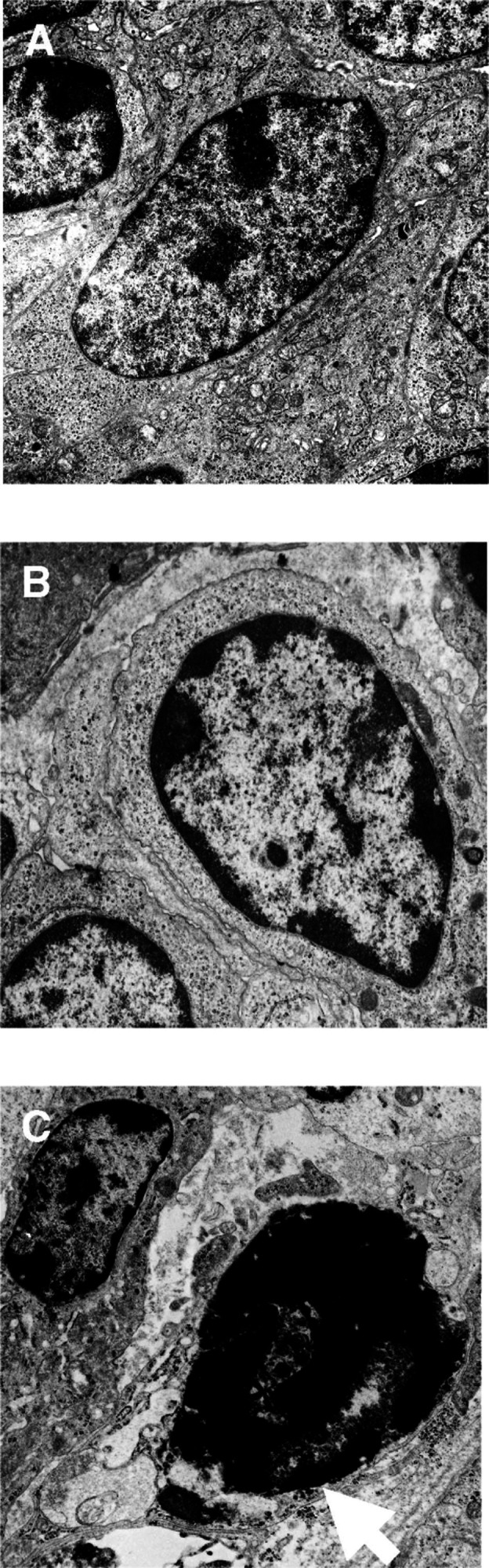
Electron microscopy images of LPB tumours treated by ECT: (**A**) untreated control fibrosarcoma; (**B**) typical apoptotic cell observed in tumours removed 30 min after ECT using 1 mg BLM; (**C**) atypical cell observed in tumours removed 25 h after ECT using 10 *μ*g BLM, the arrow indicating the double membrane; × 17 500.

Slices of B16F0 tumours treated with 1 mg of BLM and removed at 10 min, 15 min and 1 h were analysed to verify that the rapid change in chromatin morphology was similar to that observed when apoptosis occurred spontaneously or when it was induced by other known agents. Changes in the nuclei were actually typical of apoptotic cells (Figure 4A, B

): the chromatin was condensed at the limit of the nucleus, forming clumps pressed against the nuclear envelope, and the cytoplasm was dense. Moreover, at 1 h, mitochondria displayed autolytic changes (data not shown).

We also checked whether the atypical cells detected under light microscopy showed ultrastructural modifications similar to or different from those of the apoptotic cells. The chromatin fragments that are observed in atypical cells can be surrounded by a double membrane (Figure 4C) as already found by Dini *et al* (1996), who treated the cells using H_2_O_2_ and described the images seen in the atypical cells as a result of perturbations in the condensation of the mitotic chromosomes.

### Immunohistochemistry

#### TUNEL-specific assay for apoptotic cells

**Figure 5:**
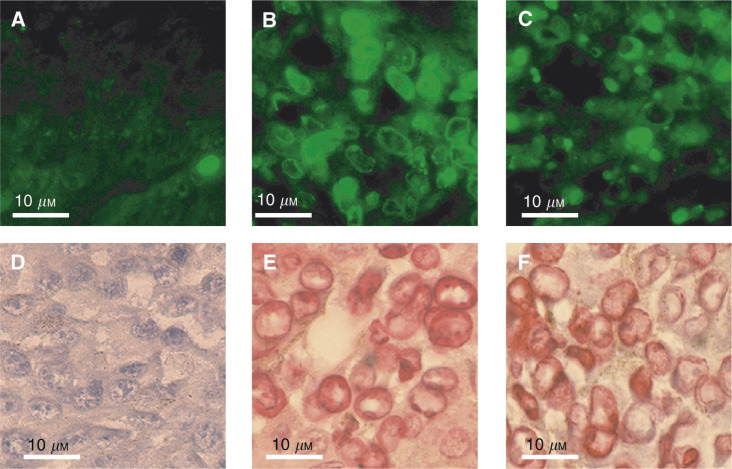
Immunohistochemical detection of DNA fragmentation in tumour cells. Fluorescein detection (**A**–**C**); alkaline phosphatase detection (**D**–**F**); (**A**–**C**): LPB fibrosarcoma; (**D**–**F**): B16F0 melanoma; (**A** and **D**): stained untreated controls; (**B** and **E**): staining of tumours removed 30 min after ECT using 1 mg BLM; (**C** and **F**): staining of tumours removed 50 h after ECT using 10 *μ*g BLM.

Nuclei presenting chromatin condensation as well as atypical cells were found positive with the TUNEL assay demonstrating that chromatin was fragmented in these cells (Figure 5

). The TUNEL staining was also applied to LPB cells electropermeabilised *in vitro* in the presence of high external concentrations of BLM (from 10 to 66 *μ*M) that resulted in pseudoapoptosis. A progressive increase in the number of positive cells and in the intensity of the staining was found with increasing external concentrations of BLM (data not shown). Therefore, the TUNEL assay was able to label both the real apoptotic cells and the pseudoapoptotic cells generated by the introduction, *in vivo*, of large amounts of BLM into the tumour cells. At a low dose of BLM, cells were TUNEL negative for at least 15 h suggesting strongly that the number of DSB directly generated by the low BLM dose was very low.

#### Caspase-3 staining

In the absence of treatment, a large number of the atypical cells found in the LPB or B16 tumours were caspase-3 positive, while only a few of the apoptotic cells were caspase-3 positive.

**Figure 6:**
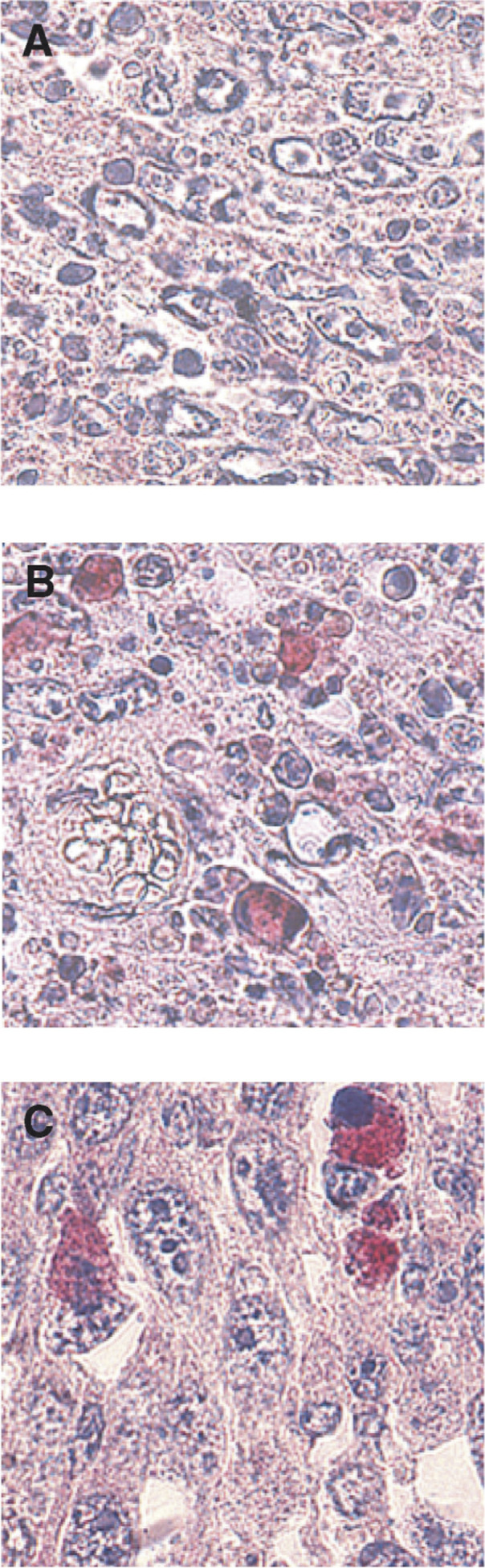
Immunohistochemical detection of activated caspase-3: (**A** and **B**): LPB tumour removed 5 h after ECT using 1 mg BLM; (**C**) LPB tumour removed 50 h after ECT using 10 *μ*g BLM.

After ECT of LPB tumours using 1 mg BLM, a large number of cells very rapidly displayed apoptotic characteristics (Figure 3), whereas the anticaspase-3 staining was restrained only to a very few apoptotic or atypical cells, confirming the pseudoapoptotic evolution suggested by the morphological analysis. At 5 h, when the percentage of LPB atypical cells was very high, the number of stained cells was also high, involving a large fraction of these atypical cells (Figure 6

).

After ECT with 10 *μ*g of BLM, caspase-3 staining was observed only after 25 h in most of the atypical cells, as well as a few of the apoptotic cells. All the large cells were caspase-3 positive and their staining was more intense than the staining observed after a high dose of BLM or in untreated controls. In additional experiments (data not shown), slides of tumours treated with an intermediary dose of BLM (100 *μ*g per injection) were also examined. Their histological analysis revealed a large number of atypical cells that were mainly caspase-3 positive, even though, again, all the atypical or condensed cells were not stained.

## DISCUSSION

Two important findings emerged from these results. First, ECT with BLM immediately induces *in vivo* a large decrease of the number of cells in mitosis and secondly, BLM induces two distinct types of tumour cell death, as a function of the BLM dose injected before the ECT. These observations can be related to previous data using cells in culture. First, *in vitro*, no mitosis is detectable after cell electropermeabilisation in the presence of BLM: cells accumulate in G_2_/M if low concentrations of BLM are used, while pseudoapoptotic figures are generated in the presence of large BLM concentrations. Secondly, *in vitro* as well (Tounekti *et al*, 1993), after cell electropermeabilisation in the presence of different doses of BLM, we observed two very different groups of morphological features that, as further discussed here, allow to conclude that, both *in vitro* and *in vivo,* tumour cells can die either through a mitotic cell death process at low BLM doses or by pseudoapoptosis at high doses. This BLM-dose-dependent cell death is present in the two tumours studied, which were chosen for this study because both tumours grow in the C57Bl/6 mice, keeping homogeneous the host reactions, and because the same pulse parameters are necessary to achieve the electropermeabilisation of almost all the cells in the tumour. Interestingly, these two tumours differ by their histological origins, spontaneous tendency to necrose and spontaneous lymphocytic infiltrate. Moreover, they respond differently to the EP (Mekid and Mir, 2000).

### Evolution of the number of ‘apoptotic figures’ and ‘atypical figures’, and origin of these figures

Besides the decrease in the number of mitotic figures, our new *in vivo* data show that, at low and high BLM doses, there is a large increase of two types of figures that we classified as ‘apoptotic figures’ and ‘atypical figures’ (already present in low percentages in the untreated tumours). For the LPB and B16F0 apoptotic cells, similar changes were observed, except that, at the high BLM dose, the increase in the number of apoptotic cells was detected earlier in the LPB than in the B16F0 tumours. The atypical figures were previously analysed after *in vitro* cell treatment by H_2_O_2_ (Shacter *et al*, 2000): according to these authors, atypical figures correspond to a delayed cell death and can be described as pycnotic/necrotic cells showing cytoplasmic swelling and mild pycnosis of the nucleus. On the one hand, after ECT, at the low BLM dose, the appearance kinetics of the atypical figures were similar for both the tumour types. They appeared after a long period (25 h), which is, nevertheless, much shorter than the *in vivo* generation time (Sersa *et al*, 1994). Since in the mean time the cells had become much larger, these atypical figures were mainly large cells. They represent a phase in the process of mitotic cell death, and as discussed below could be either a step before the appearance of the classical apoptotic figures or another step of the cells dying through the mitotic cell death pathway. On the other hand, at the high BLM dose, atypical figures appeared rapidly (in a few minutes in the LPB tumours, and at 2 h and then at 8 h in B16F0 tumours). Cells had no time to enlarge and did not differ in size from the untreated control cells. Thus, the origin of the atypical cells could be different, depending on the BLM dose.

The analysis of the atypical and the apoptotic cells shows that both are TUNEL positive. However, while most of the atypical cells were caspase-3 positive, most of the apoptotic cells were not. These two types of figures could perhaps correspond to two different stages in a common apoptotic pathway (Hadjiloucas *et al*, 2001) or to two different apoptotic cell death pathways, one caspase-3 dependent and one not (Tauchi and Sawada, 1994; Yanagihara *et al*, 1995; Kolenko *et al*, 2000). These two pathways could result from differences in the cell cycle position of the cells at the time of their electropermeabilisation and, therefore, at the time of the DSB generation, or from a different number of BLM molecules internalised (thus of a different number of DSB). It is interesting to note that using low BLM doses, the number of DSB is low just after the ECT and cells remain negative to the TUNEL assay for several hours (the DSB directly generated by BLM are actually recognised by the TUNEL assay, as reported in the Results section). However, atypical cells (as well as apoptotic cells) are TUNEL positive, indicating that a cell process generating DSB apoptosis has been actually induced after the DNA damage provoked by the low amount of the BLM.

### *In vivo* BLM causes mitotic cell death or pseudoapoptosis

Our data show that there are two cell death types observed after ECT, which can be related to the action of the BLM entering the cells *in vivo* in different amounts and thus to the generation of different numbers of DSB. The results can be compared to those found on cells in culture where it was easy to test a large number of different BLM concentrations. *In vivo* it is not easy to acquire the amount of data as it is *in vitro*, and only two different amounts of BLM (differing 100 times) injected to the mice were analysed in detail. An intermediary concentration, 100 *μ*g per mouse, was also tested (data not shown) and intermediary results were found in the time points examined confirming the dependence, discussed below, of the changes observed on the amount of injected BLM.

*In vitro,* in the presence of 10 or 100 *μ*M BLM (high doses), cells displayed a pseudoapoptotic evolution: all the apoptosis morphological and biochemical characteristics (Kerr and Searle, 1972) could be found, shortly after the ECT (as reported in the introductory section). In fact, the speed at which these changes were obtained was dependent on the BLM external concentration: the higher that concentration, and therefore the higher the amount of BLM internalised into the cells (Poddevin *et al*, 1991), the faster the changes observed (Tounekti *et al*, 1995). Thus *in vivo*, at the high BLM dose, the large numbers of rapidly appearing apoptotic figures could be the result of a pseudoapoptotic process. It is also interesting to note that, with the high BLM dose, the large amounts of apoptotic figures here reported at 30 min after ECT were, in fact, localised in defined areas of the tumours, where almost all the cells in that area displayed chromatin condensation. These areas were almost undetectable at 1 h, may be because the apoptotic residues had been phagocytosed by the neighbouring live cells. At 2 or 3 h, large areas comprising almost exclusively apoptotic figures were found again. This observation can be explained taken into account that (i) *in vitro* the speed at which chromatin condensation is detected in electropermeabilised cells exposed to high external BLM concentrations is dependent on the external concentration, (ii) the supply of BLM in the tumour is not homogeneous and very much related to the tumour vasculature and (iii) that the electric field distribution and thus cell electropermeabilisation is not totally homogeneous within the whole tumour. Thus, like *in vitro*, the larger the amount of BLM available and internalised, the faster the appearance of the apoptotic figures, suggesting that *in vivo* as well, a ‘pseudoapoptosis’ is the mechanism of cell death caused by the rapid uptake of large amounts of BLM.

For the low doses of BLM, the *in vivo* and *in vitro* evolutions are not identical. Indeed, *in vitro*, in the presence of 10 or 100 nM BLM (low doses), the electropermeabilised cells died slowly through a process reminiscent of the mitotic cell death described after the *in vitro* treatment of cells by ionising radiation (Chang and Little, 1991; Radford, 1991). No mitosis was detectable for a period equivalent to a cell cycle after the BLM internalisation, resulting in the accumulation of the cells in G_2_. This blockage was followed by an abnormal mitosis that resulted in the appearance of only one, large, often binucleated cell that could still complete a second entire cell cycle (Tounekti *et al*, 1993). A second mitosis could sometimes be observed. Karyotypes were highly modified (Tounekti *et al*, 1993). Cell death, that is, the cessation of cell metabolism, and the disappearance of cell structure occurred by processes that could not be described. Indeed, *in vitro*, the experimental synchronisation of the mitotic cell death triggering obtained by the cell electropermeabilisation, which allowed the study of the initial steps of this pathway, could not be maintained to describe its final steps. We could not detect the appearance of the oligonucleosomal ladder, but this does not mean that no apoptosis could occur. In fact, we supposed that cells were dying because of necrosis (i.e. that cells swelled, lysed and released their content into the extracellular medium).

*In vivo*, at low BLM doses, cells present an evolution that, at the beginning, is reminiscent of the *in vitro* observations (transient mitosis disappearance). However, *in vivo*, the end point of this evolution seems to be typical and/or ‘atypical’ apoptosis. It cannot be totally excluded that the atypical cells could be the result of an ‘atypical apoptosis’. However, in agreement with the conclusions of Hadjiloucas *et al* (2001), our results suggest that *in vivo* the atypical cells, which are caspase-3 positive, could be an intermediary step between the still alive treated cells and the apoptotic dead cells. Caspase-3 positive staining, which allows to distinguish easily the atypical cells generated during the mitotic cell death process, is thus an interesting early marker of apoptosis, as already suggested by Hadjiloucas *et al* (2001).

There is still another possibility to explain the differences between the *in vitro* and *in vivo* results using small doses of BLM. Indeed, in the B16 tumours, no major change in the immune system infiltration was detected, while in the LPB tumours, after ECT with 10 *μ*g BLM, a massive infiltration of immune system cells was observed at the time points where a large fraction of apoptotic cells was also detectable (25–100 h after the treatment). Interestingly, the percentage of apoptotic cells in LPB tumours was slightly higher than that in the B16F0 tumours. Of course, this could result from different kinetics in the generation and disappearance of the apoptotic cells in both tumours, but it cannot be excluded that the infiltrating immune system cells present in the LPB tumours at these time points could contribute to the generation of apoptotic cells. As a matter of fact, it is known that LPB cells are immunogeneic (Belehradek *et al*, 1972) while the B16F0 are not. In parallel, it must be noted that a large fraction (up to 60%) of LPB tumours can be cured after one single ECT session using 10 *μ*g of BLM per mice (Mir *et al*, 1992) while, using exactly the same protocol, only 16% of the B16F0 tumours could be cured (Sersa *et al*, 1995). Moreover, the observation (Figure 2B) of the presence of a small fraction of mitotic B16F0 cells in the treated tumours (almost not detected in the LPB tumours) from 30 h on after the ECT is quite in line with the results of tumour cure. Thus, after the injection of a low BLM amount, the apoptosis detected in B16F0 tumours reinforces the suggestion that, *in vivo,* the final outcome of mitotic cell death is an apoptosis, in agreement with previous *in vitro* studies using ionising radiation (Tauchi and Sawada, 1994; Yanagihara *et al*, 1995). Simultaneously, our results also suggest that in LPB tumours the high fraction of apoptotic cells could result from the direct outcome of the treatment and the concomitant cytotoxic activity of the immune system.

In summary, using the high BLM dose, a pseudoapoptotic evolution of the tumour cells was found, reminiscent of the observation performed *in vitro* on cells electropermeabilised in the presence of high external BLM concentrations. Using the low dose, a cell death pathway like the one observed after tumour treatment by ionising irradiation (mitotic cell death) was observed shortly after the treatment. This cell death pathway ended through a real apoptotic process. The atypical caspase-3 positive figures are an intermediary step in this process. Thus, antiactivated caspase-3 staining is an early marker of apoptosis. Moreover, the difference in the results obtained *in vivo* with the high and low amounts of BLM can actually be interpreted, as *in vitro*, as the direct consequence of the number of BLM molecules internalised into the cells and, therefore, as the result of the number of DSB generated in these cells.
